# Evaluating the Effectiveness of Trauma Care and Emergency Preparedness Training Programs on Prehospital Primary Survey Skills: A Systematic Review

**DOI:** 10.7759/cureus.74089

**Published:** 2024-11-20

**Authors:** Abdullah Sajid, Areej Shakir, Manahil Awan, Fnu Warsha, Shahzad Ahmad, Lara Alsadoun, Muhammad Qaiser Aziz

**Affiliations:** 1 Cardiology, Pervaiz Elahi Institute of Cardiology, Bahawalpur, PAK; 2 Intensive Care, Fatimiyah Hospital, Karachi, PAK; 3 General Medicine, Liaquat National Hospital, Karachi, PAK; 4 Internal Medicine, Norton Community Hospital, Norton, USA; 5 Cardiac Surgery, Liaquat National Hospital, Karachi, PAK; 6 Trauma and Orthopedics, Chelsea and Westminster Hospital, London, GBR

**Keywords:** emergancy, prehospital training, primary survey, skill training, trauma care

## Abstract

This systematic review evaluates the impact of trauma care and emergency preparedness training programs on prehospital primary survey effectiveness. A comprehensive search strategy was employed across multiple databases, including PubMed, Cochrane Library, Embase, and the Cumulated Index to Nursing and Allied Health Literature (CINAHL), focusing on studies involving healthcare professionals such as paramedics, nurses, and emergency medical technicians (EMTs). The review included randomized controlled trials (RCTs), clinical trials, and cohort studies that assessed various training modalities like virtual reality (VR) simulations, case-based learning (CBL), and hands-on workshops. Quality assessment was performed using the Cochrane risk-of-bias (RoB) tool for randomized trials and the Newcastle-Ottawa Scale (NOS) for clinical trials, ensuring methodological rigor and consistency. The findings suggest that CBL significantly improves knowledge retention and prehospital primary survey skills, outperforming other methods such as simulation exercises, which showed mixed results. VR training increased confidence levels but did not demonstrate significant improvements in objective skills compared to traditional methods. The use of supplementary triage assistance teams (physician-nurse supplementary triage team (MDRNSTAT)) was found to be effective during high patient volume hours, though not cost-effective as a daytime strategy. While the review highlights the importance of interactive and scenario-based training programs, limitations such as variability in study designs, publication bias, and language bias were noted, suggesting that caution should be exercised in generalizing the results. Future research should focus on long-term effectiveness, the integration of emerging technologies, and larger, well-designed trials across diverse healthcare settings to strengthen the evidence base.

## Introduction and background

Trauma care and emergency preparedness training programs play a critical role in enhancing the skills of healthcare providers, particularly in the prehospital setting, where rapid and effective intervention can significantly impact patient outcomes [1]. The effectiveness of these programs, especially in training providers on conducting a primary survey, a systematic approach used to quickly assess and manage life-threatening conditions, has become a focus of research and development [2]. A primary survey is the foundation of trauma care and involves a structured evaluation of airway, breathing, circulation, disability, and exposure (ABCDE) to stabilize patients during emergencies [3,4]. As an integral outcome measure, the primary survey allows for the assessment of training effectiveness by determining the proficiency of healthcare providers in identifying and managing life-threatening conditions such as airway obstruction, tension pneumothorax, or uncontrolled hemorrhage immediately. Training healthcare providers, including paramedics, nurses, and physicians, in these critical skills is essential to improve trauma care delivery, particularly in resource-limited settings and high-risk environments such as disaster zones and road traffic accidents [5]. As advancements in training modalities, including virtual reality (VR) simulations and case-based learning (CBL), emerge, it is essential to evaluate the effectiveness of these methods to determine best practices and optimize training programs for healthcare professionals.

To systematically assess the impact of trauma care and emergency preparedness training programs on the effectiveness of prehospital primary survey skills, the population, intervention, comparison, and outcome (PICO) framework is utilized [6]. The population (P) for this review includes healthcare professionals such as paramedics, nurses, and emergency medical technicians (EMTs) who are actively involved in prehospital trauma care. The intervention (I) focuses on trauma care and emergency preparedness training programs designed to improve prehospital primary survey skills, incorporating various training modalities such as workshops, VR simulations, and CBL. The comparison (C) is made against standard training methods or the absence of formal training programs. The outcome (O) being measured is the improvement in prehospital primary survey skills, which encompasses proficiency in airway management, breathing assessment, circulation control, disability evaluation, and overall trauma management effectiveness. This PICO framework serves as a guide for the systematic review, aiming to evaluate how these training programs enhance the knowledge, skills, and preparedness of healthcare providers. Furthermore, it seeks to identify the most effective methods for delivering these training programs to optimize the skill development of healthcare professionals in prehospital trauma care settings.

The primary objective of this systematic review is to evaluate the impact of trauma care and emergency preparedness training programs on the effectiveness of prehospital primary survey skills among healthcare professionals. Effectiveness is defined operationally as the measurable improvement in knowledge retention, procedural skill proficiency, confidence levels, and objective patient management outcomes, as assessed through validated tools such as pre- and post-training knowledge tests, simulation scores, and real-life application success rates. By synthesizing evidence from diverse training methods, including workshops, VR simulations, and CBL, this review aims to identify best practices and highlight gaps in existing programs, focusing on key metrics like knowledge enhancement, skill acquisition, and clinical application. The review will also explore the outcomes associated with different training approaches to determine which methods result in the most significant improvements in prehospital trauma care delivery. Ultimately, the goal is to provide recommendations for optimizing training programs to enhance the skills of healthcare providers, thereby improving patient outcomes in emergency and trauma settings.

## Review

Materials and methods

Search Strategy

To conduct a comprehensive systematic review evaluating the impact of trauma care and emergency preparedness training programs on prehospital primary survey effectiveness, a robust and detailed search strategy was employed. The search was carried out across multiple electronic databases, including PubMed, Cochrane Library, Embase, and the Cumulated Index to Nursing and Allied Health Literature (CINAHL), to ensure a wide coverage of relevant studies. Keywords and Medical Subject Headings (MeSH) terms were used in combination to refine the search, such as "prehospital primary survey," "trauma care training," "emergency preparedness," "disaster response education," and "paramedic training." Boolean operators like "AND" and "OR" were applied to optimize the search results and capture studies focusing on different training modalities, including VR simulations, CBL, and hands-on workshops. The search was restricted to articles published in English from the year 2000 onward to ensure the inclusion of recent evidence, reflecting modern training programs and technology advancements in trauma education.

Additionally, reference lists of identified studies were reviewed for any additional relevant articles that may not have appeared in the initial database search, a process known as citation chaining. Grey literature sources such as conference proceedings, reports, and theses were also explored to identify unpublished data and minimize publication bias. The search was iterative, with repeated refinement of search terms and strategies based on preliminary results, ensuring the inclusion of all relevant studies meeting the inclusion criteria. The selected articles were screened for eligibility based on predefined criteria, including study design (randomized controlled trials (RCTs), clinical trials), population (healthcare professionals involved in prehospital trauma care), and outcomes (effectiveness in prehospital primary survey skills). This comprehensive approach ensured a thorough and systematic identification of relevant studies for inclusion in the review, which was conducted in accordance with the Preferred Reporting Items for Systematic Reviews and Meta-Analyses (PRISMA) guidelines.

Eligibility Criteria

The eligibility criteria for this systematic review were defined to ensure the inclusion of high-quality studies that specifically address the impact of trauma care and emergency preparedness training programs on prehospital primary survey effectiveness. Studies were included if they were RCTs, clinical trials, or cohort studies that evaluated training interventions aimed at healthcare professionals, such as paramedics, nurses, EMTs, or advanced medical students involved in prehospital trauma care. The interventions of interest included various training modalities, such as VR simulations, CBL, hands-on workshops, and supplementary triage assistance programs, all designed to enhance skills in conducting a primary survey in emergency or disaster scenarios. Studies were required to measure outcomes directly related to prehospital primary survey effectiveness, such as knowledge attainment, skill proficiency, patient management, and objective assessments of trauma care skills.

Exclusion criteria were also applied to filter out studies that did not meet the focus of the review. Studies that involved non-healthcare professional populations or those not specifically engaged in prehospital trauma care, such as general disaster preparedness courses for non-medical personnel, were excluded. Additionally, studies published in languages other than English or those published before the year 2000 were not included, as the review aimed to focus on recent and relevant evidence reflecting modern training techniques and advancements in trauma education. Studies that lacked control or comparison groups or that did not provide measurable outcomes related to the primary survey skills were also excluded to maintain the rigor and relevance of the review. This clear and focused eligibility criterion ensured that the systematic review included only high-quality evidence that directly informs the effectiveness of trauma care training programs in the prehospital setting.

Data Extraction

The data extraction process for this systematic review was designed with a structured and standardized approach to ensure consistency and accuracy in capturing relevant information from each included study. A data extraction form was developed and piloted based on the objectives of the review and the PICO framework. This form included essential details such as study design, population characteristics, intervention type, comparison group, outcome measures, results, and statistical data. Key data points, such as knowledge scores, skill proficiency metrics, confidence levels, and effect sizes, were determined based on their relevance to assessing the effectiveness of trauma care training programs and prehospital primary survey skills, as defined in the inclusion criteria. These data points were selected through consensus among the review team and informed by established literature on trauma training evaluation metrics. Two independent reviewers extracted data from each study to minimize bias, with discrepancies resolved through discussion or consultation with a third reviewer. Special emphasis was placed on extracting data related to primary survey skills, training modalities (e.g., VR simulations, CBL), and outcome assessment methodologies. This comprehensive and systematic approach ensured that all relevant aspects of each study were captured, facilitating a thorough and reliable analysis of evidence for the review.

Data Analysis and Synthesis

For data analysis and synthesis in this systematic review, a narrative synthesis approach was employed to summarize and integrate findings from the included studies, given the diversity in intervention types and outcome measures. The extracted data were systematically reviewed to identify patterns, similarities, and discrepancies across studies, focusing on the effectiveness of various trauma care and emergency preparedness training programs in enhancing prehospital primary survey skills. Studies were grouped according to the type of intervention (e.g., VR simulations, CBL, hands-on workshops) and their measured outcomes, such as improvements in knowledge scores, procedural skills, and confidence levels. The analysis emphasized the quality and robustness of the evidence, incorporating risk-of-bias (RoB) assessments to evaluate the reliability of findings. Effect sizes, where available, were qualitatively compared to highlight the relative impact of different training modalities. This structured narrative synthesis allowed for a comprehensive summary of the evidence, facilitating conclusions about the most effective training programs and identifying areas for future research. 

Results

Study Selection Process

**Figure 1:**
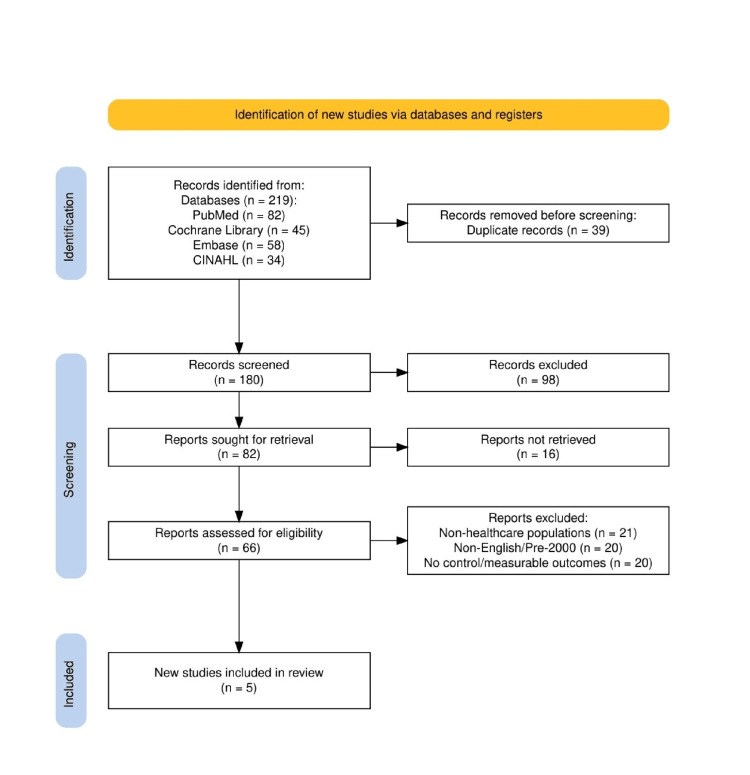
The PRISMA flowchart represents the study selection process PRISMA: Preferred Reporting Items for Systematic Reviews and Meta-Analyses

The study selection process began with a comprehensive search across four databases: PubMed, Cochrane Library, Embase, and CINAHL, yielding a total of 219 records. After removing 39 duplicate records, 180 unique studies were screened based on their titles and abstracts. Out of these, 98 records were excluded due to irrelevance to the review focus. For the remaining 82 reports, full-text retrieval was attempted; however, 16 reports could not be obtained. The remaining 66 reports were then assessed for eligibility based on predefined criteria, resulting in the exclusion of 61 reports for reasons such as involving non-healthcare populations (21), being non-English or published before 2000 (20), or lacking control groups/measurable outcomes (20). Ultimately, five studies met the inclusion criteria and were incorporated into the review for detailed analysis. The following PRISMA flow diagram (Figure 1) illustrates the selection process in detail, outlining the number of studies identified, screened, assessed for eligibility, and ultimately included in the review. This diagram provides a clear visual representation of the systematic review process, following PRISMA guidelines for transparency and reproducibility.

Characteristics of the Selected Studies

The characteristics of the selected studies provide a diverse representation of trauma care and emergency preparedness training programs aimed at healthcare professionals. The studies included both RCTs and clinical trials, focusing on different healthcare settings and populations, ranging from nursing trainees and advanced medical students to paramedics and medical staff in tertiary care environments. Interventions varied across the studies, including CBL, VR simulations, supplementary triage teams, and hands-on trauma care workshops. Comparisons were made between these interventions and traditional methods, such as standard didactic instruction or nurse-only triage. Outcome measures primarily assessed the effectiveness of these training programs in improving knowledge scores, procedural skills, patient management efficiency, and satisfaction levels. The results highlighted both the effectiveness and limitations of these interventions, with statistical data demonstrating significant improvements in some cases, such as CBL’s impact on knowledge retention and VR's influence on confidence levels. Additionally, the findings emphasized the need for strategic implementation and continuous training for sustained effectiveness. A detailed summary of the selected studies is provided in Table 1, which outlines key characteristics, methodologies, and outcomes of each study included in the review.

**Table 1:** Summary of characteristics and findings of selected studies on trauma care and emergency preparedness training programs ED: emergency department; ATLS: advanced trauma life support; EMS: emergency medical services; EDLOS: emergency department length of stay

Study reference	Study design	Population	Intervention	Comparison	Outcome measures	Results/key findings	Statistical data
Cheng et al., 2016 [7]	Randomized controlled trial (RCT)	Patients and healthcare professionals (physician-nurse team) in an academic tertiary care ED	Physician-nurse supplementary triage assistance team (MDRNSTAT)	Nurse-only triage	Cost per additional patient seen, cost per physician initial assessment (PIA) hour saved, cost per ED length of stay (EDLOS) hour saved, patient satisfaction	MDRNSTAT added $3,597.27 per additional patient seen, $75.37 per PIA hour saved, $112.99 per EDLOS hour saved; not cost-effective as a daytime strategy but feasible from noon to midnight for higher patient volumes	Incremental cost-effectiveness ratio: $3,597.27 per patient, $75.37 per PIA hour, $112.99 per EDLOS hour; cost-benefit ratio (hospital): 38.6; cost-benefit ratio (patient satisfaction): 2.8
Aluisio et al., 2016 [8]	RCT	60 nursing trainees in disaster preparedness education in Lucknow, India	Case-based learning (CBL) added to standard didactic instruction	Simulation exercises (SEs) added to standard didactic instruction	Knowledge attainment scores pertaining to disaster triage preparedness	CBL resulted in a significant increase in knowledge scores (20.8% intergroup, 10.3% intragroup; p = 0.003 and p = 0.033, respectively). SEs did not significantly improve scores (6.6% increase; p = 0.396) and even showed a decrement in crossover analysis (26.0% reduction; p < .001).	CBL vs. SE: p = 0.003 (intergroup), p = 0.033 (intragroup); SE decrement: 26.0%, p < .001
Amiri et al., 2013 [9]	Clinical trial	64 medical staff participants attending the primary trauma care (PTC) workshop at Tabriz Medical University	Two-day primary trauma care workshop, including lectures, hands-on practices, and case scenarios	None	Pretest and post-test scores (early and late) on trauma care knowledge and procedural skills	Mean scores improved from 18.84 (pretest) to 26.72 (early post-test) and decreased to 22.17 (late post-test) (p < 0.001). Demonstrates the effectiveness of the workshop initially but highlights the need for further training to maintain skills over time	Mean score changes: pretest = 18.84, early post-test = 26.72, late post-test = 22.17; p < 0.001
Birrenbach et al., 2024 [10]	RCT	56 advanced medical students taking part in the ATLS course at the Military Physician Officer School	Virtual reality (VR) trauma simulation as adjunct training for ATLS course	Traditional ATLS training only	Feasibility, objective effectiveness (clinical scenario scores, knowledge tests, pass rates), subjective effectiveness (confidence ratings), acceptance (usability, presence, immersion)	VR training demonstrated high feasibility with only one premature termination. No significant differences in objective measures (knowledge tests, scenario scores, pass rates) between groups; however, significant improvement in confidence post-VR intervention (p < .001). High acceptance and usability scores (mean 79.4 on the System Usability Scale)	Objective effectiveness: p = 0.832 (pretest), p = 0.237 (final test), p = 0.485 (pass rates); subjective effectiveness (confidence): p < .001; usability mean score: 79.4 (SD 11.3)
Spaite et al., 2001 [11]	Clinical trial	Paramedics and children (21 years or younger) with special healthcare needs	Specialized paramedic training focusing on the assessment and management of children with special healthcare needs	Pretraining EMS responses	Appropriate assessment and management rates, inter-reviewer agreement, improvement in paramedic assessment skills	Significant improvement in appropriate assessment and overall care by paramedics post-training. High agreement (>70%) among reviewers for most items, although kappa statistics varied	Inter-reviewer agreement ranged from 32% to 93%. High agreement (>70%) between nurses and physicians on most items; kappa statistics generally did not indicate good agreement except for focused assessment and some treatment items

Quality of the Selected Studies

The overall quality of the selected studies is moderate to good, as evaluated using the Cochrane RoB Tool for RCTs and the Newcastle-Ottawa Scale (NOS) for clinical trials. Most studies demonstrated low RoB in key domains such as random sequence generation, allocation concealment, and outcome assessment. However, challenges in blinding of participants and personnel were noted, particularly in studies where blinding was not feasible, resulting in moderate performance bias. Clinical trials assessed with the NOS showed strong selection criteria and adequate outcome assessments, although some lacked comparability due to the absence of control groups. Despite these limitations, the majority of studies maintained a good overall quality, supporting the reliability of their findings, presented in Table 2.

**Table 2:** Quality assessment of selected studies

Study reference	Study design	Quality assessment tool	Domains assessed	Assessment results
Cheng et al., 2016 [7]	Randomized controlled trial (RCT)	Cochrane risk-of-bias (RoB) tool	Random sequence generation, allocation concealment, blinding of participants/personnel, outcome assessment, incomplete outcome data, selective reporting, other bias	Low RoB in random sequence generation and allocation concealment. High risk in blinding of participants/personnel. Moderate risk in outcome assessment due to lack of blinding. Low risk for other domains
Aluisio et al., 2016 [8]	RCT	Cochrane RoB tool	Random sequence generation, allocation concealment, blinding of participants/personnel, outcome assessment, incomplete outcome data, selective reporting, other bias	Low RoB across most domains except for blinding of participants/personnel, which showed a moderate risk. The outcome assessment was blinded, reducing detection bias. Incomplete data were reported comprehensively
Amiri et al., 2013 [9]	Clinical trial	Newcastle-Ottawa Scale (NOS)	Selection of study groups, comparability of groups, outcome assessment	Good selection of study groups and adequate outcome assessment. Limited comparability due to lack of control group beyond pre/post comparison
Birrenbach et al., 2024 [10]	RCT	Cochrane RoB tool	Random sequence generation, allocation concealment, blinding of participants/personnel, outcome assessment, incomplete outcome data, selective reporting, other bias	Low RoB in sequence generation and allocation concealment. Moderate risk in performance bias (blinding of participants not possible). Outcome assessment was well blinded, and complete outcome data were reported
Spaite et al., 2001 [11]	Clinical trial	NOS	Selection of study groups, comparability of groups, outcome assessment	Strong selection criteria and good outcome assessment. Limited comparability due to the absence of a control group beyond pre-/post-intervention comparison

Discussion

The findings across the studies demonstrate varying degrees of effectiveness for trauma care and emergency preparedness training programs, with notable implications for prehospital primary survey skills. Cheng et al. [7] evaluated the cost-effectiveness of the physician-nurse supplementary triage team (MDRNSTAT) and found that, while it provided some benefits, it was not a cost-effective daytime strategy. The incremental cost-effectiveness ratio highlighted the high costs per patient and per physician initial assessment (PIA) hour saved, suggesting that the intervention may only be viable during peak patient volume periods. This emphasizes the need for strategic timing and allocation of such interventions in emergency departments to maximize efficiency. In contrast, Aluisio et al. [8] showed that CBL significantly improved knowledge attainment scores compared to simulation exercises (SEs) in disaster preparedness education for nursing trainees. The statistically significant improvements in knowledge scores (p = 0.003 intergroup and p = 0.033 intragroup) indicate that CBL could be a more effective modality for similar training programs, while the decrement in knowledge retention observed in SEs (26% reduction; p < .001) suggests that not all simulation-based approaches yield positive outcomes.

The study by Amiri et al. [9] demonstrated the effectiveness of a two-day primary trauma care workshop, with significant improvements in knowledge and procedural skills immediately after the training (p < 0.001). However, the decline in scores in the late post-test highlights a challenge in maintaining skills over time, indicating a need for continuous or repeated training to reinforce knowledge retention. Similarly, Birrenbach et al. [10] assessed a VR trauma simulation and found it to be a feasible and accepted adjunct in advanced trauma life support (ATLS) courses, with participants showing a significant increase in confidence levels (p < .001) despite no significant changes in objective measures such as test scores and pass rates. Spaite et al. [11] also highlighted the benefits of specialized training for paramedics in managing children with special healthcare needs, showing improvements in appropriate assessment and care, though inter-reviewer agreement varied. These findings suggest that while certain training modalities demonstrate measurable short-term gains, long-term effectiveness and knowledge retention remain critical areas for further research, particularly in identifying the most impactful training formats and intervals to sustain skills over time.

The findings of this systematic review align with and expand upon existing literature on trauma care and emergency preparedness training programs [12]. Similar to the results found in Aluisio et al. [8], previous studies have also demonstrated that CBL is a highly effective modality for knowledge retention and skill acquisition, particularly in disaster preparedness and emergency response training [13]. This is consistent with prior evidence showing that interactive and scenario-based learning approaches engage learners more effectively compared to traditional didactic or simulation exercises, which may not always mirror real-life complexities adequately [14]. The significant improvement in knowledge scores observed in our review reaffirms the potential of CBL as a superior method for enhancing prehospital primary survey skills. In contrast, simulation-based learning, while popular in the literature, has shown mixed results; some studies have reported improvements in procedural skills, but others, including this review, indicate limitations in knowledge retention and effectiveness, particularly in high-pressure or diverse scenarios [15].

This review also provides new insights into the application of VR and supplementary triage assistance teams (such as the MDRNSTAT) in trauma care education. Previous studies have mostly focused on VR for surgical or technical skills, but the results here suggest that while VR can enhance learner confidence, its impact on objective skills and test performance remains limited [16]. This discrepancy suggests that while VR technology is promising, it may need further refinement and integration with other training methods to be fully effective for trauma and emergency preparedness. Additionally, the analysis of the MDRNSTAT intervention offers a novel perspective on the cost-effectiveness of multiprofessional training teams [7]. The findings challenge the assumption that these teams are universally beneficial, emphasizing the importance of context (such as peak patient volume) when implementing such interventions. These insights contribute to the field by highlighting the need for a more strategic and tailored approach to trauma education, integrating the strengths of each training modality for optimal outcomes [17].

The findings of this systematic review have significant implications for healthcare practice, education, and policy, particularly in optimizing trauma care and emergency preparedness training programs. For healthcare professionals and educators, the evidence suggests that integrating CBL into existing training frameworks could enhance knowledge retention and improve the effectiveness of prehospital primary survey skills, as it has proven to be more impactful than simulation exercises [18]. Policymakers should consider these results when designing and funding trauma education programs, emphasizing interactive and scenario-based training approaches that mirror real-life situations more closely. Furthermore, the evidence on VR suggests that while it increases confidence, its effectiveness in improving objective skills is limited unless supplemented with additional hands-on or case-based components. Therefore, incorporating VR as a supplementary tool rather than a standalone method may enhance training outcomes. The insights on the MDRNSTAT highlight the need for strategic implementation, recommending its use during peak hours to maximize efficiency and cost-effectiveness. Overall, these findings can guide the development of comprehensive, evidence-based training programs that are adaptive and resource-efficient, ultimately improving trauma care outcomes.

This systematic review has several strengths, including a comprehensive search strategy that spanned multiple databases and sources, ensuring a broad capture of relevant studies. The use of robust quality assessment tools, such as the Cochrane RoB tool [19] and the NOS [20], allowed for consistent and thorough evaluation of study quality, enhancing the validity and reliability of the findings. Additionally, the consistency in data extraction and synthesis processes helped maintain methodological rigor, and the adherence to PRISMA guidelines [21] ensured a structured and transparent approach. These measures collectively ensure that the conclusions drawn are based on systematically evaluated evidence from high-quality studies. However, the review also has limitations. The variability in study designs and heterogeneity in interventions and outcome measures made direct comparisons challenging, potentially affecting the uniformity of the results. Furthermore, the exclusion of non-English studies could introduce language bias, and the focus on published literature might result in publication bias, as unpublished or negative studies may have been overlooked. These limitations suggest that while the findings provide valuable insights, caution should be exercised in generalizing the results across different healthcare settings or populations.

Future research should focus on addressing the gaps identified in this review, particularly the need for studies evaluating the long-term effectiveness of trauma care and emergency preparedness training programs [22]. While short-term improvements in knowledge and skills were observed, there is a lack of evidence on how well these skills are retained over time and their impact on actual patient outcomes in real-life emergencies. Additionally, further research is needed to explore the potential of emerging technologies like VR in trauma care training. While VR shows promise in increasing confidence, its effectiveness in enhancing practical skills remains inconclusive; thus, studies should investigate its integration with other modalities such as hands-on workshops and CBL [23,24]. Larger, well-designed RCTs across diverse healthcare settings and populations are also necessary to strengthen the evidence base, ensuring that findings are generalizable and applicable to various contexts, including resource-limited settings and high-volume emergency departments.

## Conclusions

This systematic review underscores the importance of well-designed trauma care and emergency preparedness training programs in enhancing prehospital primary survey skills among healthcare professionals. Interactive methods, like CBL, prove more effective for knowledge retention and skill proficiency than traditional simulations. While VR shows potential as a supplementary tool, it may not suffice as a standalone intervention for practical skills. The review also highlights the need for strategic implementation of programs like MDRNSTAT to maximize cost-effectiveness during peak emergency hours. Despite valuable insights, gaps in the long-term effectiveness of training programs call for further research. By refining evidence-based training approaches to specific healthcare settings, trauma education programs can better improve prehospital care and patient outcomes in emergencies.
